# Epidemiological Impact of Nirsevimab on Admissions for Bronchiolitis in a Pediatric Emergency Department: A Single-Center Cohort Study

**DOI:** 10.3390/v18040469

**Published:** 2026-04-16

**Authors:** Emanuele Castagno, Carola Aschieri, Irene Ferri, Sara El Khbazi, Lorenzo Milani, Rosanna Irene Comoretto, Irene Raffaldi, Irene Tardivo, Marco Spada, Claudia Bondone, Franca Fagioli

**Affiliations:** 1 Department of Pediatric Emergency, Regina Margherita Children’s Hospital, 10126 Turin, Italy; ecastagno@cittadellasalute.to.it (E.C.); cbondone@cittadellasalute.to.it (C.B.); 2 Department of Pediatrics and Public Health Sciences, Postgraduate School of Pediatrics, University of Turin, 10126 Turin, Italy; carola.aschieri@unito.it (C.A.); irene.ferri@unito.it (I.F.); 3 Department of Pediatrics, Regina Margherita Children’s Hospital, University of Turin, 10126 Turin, Italy; sara.elkhbazi@edu.unito.it (S.E.K.); itardivo@cittadellasalute.to.it (I.T.); marco.spada@unito.it (M.S.); 4 Department of Clinical and Biological Sciences, University of Turin, 10043 Orbassano, Italy; lorenzo.milani@unito.it; 5 Department of Pediatrics and Public Health Sciences, University of Turin, 10126 Turin, Italy; rosannairene.comoretto@unito.it (R.I.C.); franca.fagioli@unito.it (F.F.); 6 Pediatric Onco-Hematology, Stem Cell Transplantation and Cellular Therapy Division, Regina Margherita Children’s Hospital, 10126 Turin, Italy

**Keywords:** *Respiratory syncytial virus*, bronchiolitis, nirsevimab, infants, emergency department

## Abstract

*Respiratory syncytial virus* (RSV) is the leading cause of bronchiolitis in children < 24 months and a major public health concern, causing high rates of Emergency Department (ED) visits, hospitalizations, and Pediatric Intensive Care Unit (PICU) admissions. Nirsevimab is a recombinant monoclonal antibody recommended for all infants and high-risk children < 24 months. A retrospective single-center cohort study was conducted to evaluate the impact of nirsevimab introduction on bronchiolitis epidemiology in an Italian tertiary pediatric ED, accounting for 40,000 admissions/year. All children < 24 months who presented to our ED with bronchiolitis during two consecutive RSV seasons (first season: 1 October 2023 to 30 April 2024; second season: 1 October 2024 to 30 April 2025) were included. Descriptive and multivariate analyses are reported. Overall, 484 patients were analyzed (336 in 2023–2024; 148 in 2024–2025), with immunization coverage reaching 87.5% by April 2025. Compared with the previous season, RSV positivity decreased significantly (32.4% vs. 47.9%; *p* = 0.003) and was lower in immunized children (16.2% vs. 51.5%; *p* < 0.001). Immunization was associated with a reduced risk of RSV-positive swab in the second season (OR = 0.159, 95% CI: 0.059–0.397). Among RSV-negative patients, other respiratory viruses increased (*p* < 0.001), while co-infections increased in RSV-positive cases (*p* = 0.021). Hospitalization rates remained stable, though absolute admissions were halved. In conclusion, nirsevimab immunization reduced RSV burden, supporting its inclusion in universal prevention programs and the need for multicenter prospective studies to assess long-term outcomes.

## 1. Introduction

Bronchiolitis is an acute viral infection of the lower respiratory tract, predominantly affecting children under 2 years of age, with peak incidence between 3 and 6 months [1]. It accounts for up to 15–17% of hospitalizations in children < 2 years and 15% of pediatric Emergency Department (ED) visits [2].

*Respiratory syncytial virus* (RSV) is the predominant etiological agent, accounting for 60–80% of bronchiolitis cases and for 45–54% of admissions in infants under 6 months [2,3]. RSV is an RNA virus of the genus *Orthopneumovirus* (family *Pneumoviridae*) that is ubiquitous worldwide [4], with two major antigenic subgroups (RSV-A and RSV-B); RSV-A is often associated with higher morbidity [5]. Its error-prone replication leads to continuous antigenic drift, complicating vaccine and antiviral development [6].

RSV typically causes seasonal outbreaks in the Northern Hemisphere from October to mid-April, peaking in December–January [7]. The COVID-19 pandemic, through the implementation of non-pharmaceutical interventions, temporarily suppressed RSV circulation, but subsequent relaxation of these measures led to atypical epidemic rebounds and altered seasonality [8,9,10,11]. The epidemiological impact of RSV is significant as it is estimated to be responsible for 33 million cases of lower respiratory infection in children younger than 5-years-old annually, leading to 3.2 million hospitalizations and more than 100,000 deaths each year worldwide [12,13].

Palivizumab is a monoclonal antibody targeting the RSV fusion protein and was the only prevention option until recently. Approved in 1998, it was administered monthly during RSV season, mostly to preterm infants or those with chronic lung or congenital heart disease. Despite efficacy in high-risk populations, its limited indications and monthly dosing restrict public health utility [14].

These limitations prompted the development of nirsevimab, a long-acting recombinant human monoclonal antibody with extended half-life. Nirsevimab targets a highly conserved epitope of the prefusion F protein, blocking viral entry and cell-to-cell spread. The MEDLEY trial, a randomized multicenter phase 2/3 study, compared nirsevimab with palivizumab in high-risk infants. A single dose of nirsevimab achieved protective serum levels for approximately five to six months, thus covering a full RSV season, with similar rates of serious adverse events compared to palivizumab [15,16].

Nirsevimab was approved by the United States Food and Drug Administration in July 2023 and recommended for infants entering or during their first RSV season, as well as for high-risk children up to 24 months of age [17]. Approval was supported by two pivotal phase 3 trials: the MELODY study, which showed a significant reduction in RSV-associated lower respiratory tract infections in healthy infants [18], and the HARMONY study, which confirmed safety and efficacy across diverse pediatric populations [19].

The global rollout marked a shift toward universal RSV prevention strategies, with national immunization campaigns implemented worldwide during the 2023–2024 season. France implemented RSV immunization with nirsevimab in September 2023; Carbajal et al. reported 47% reduction in pediatric ED visits for all-cause bronchiolitis and a 83% reduction for RSV-associated cases [20]. Spain also implemented early programs, with regional vaccine uptake ranging from 84% to 95%, with nirsevimab effectiveness estimated around 74–75% in preventing RSV hospitalizations in <1-year-old infants [21].

Italy began regional implementation in late 2023, with the Valle d’Aosta region introducing universal prophylaxis. Early results showed that the risk of hospitalization was 8.3% among infants who did not receive prophylaxis, whereas none of the infants who received nirsevimab were hospitalized for bronchiolitis, with no severe adverse events reported [22]. In the rest of the country, the preventive campaign started in November 2024, offering free voluntary immunization for all infants born in 2024. Nirsevimab was offered directly in hospital birth centers to all the infants born between November 2024 and March 2025, and to infants born earlier in 2024 (January–October) through primary pediatric health services. In the Piedmont region, 71.5% eligible patients received immunization by the end of March 2025 [22].

In this context, our study aimed to evaluate the epidemiological impact of nirsevimab immunization on bronchiolitis by comparing data from the 2024–2025 RSV season, when nirsevimab was introduced, to the previous season. Our main aim was to assess the effectiveness of nirsevimab in preventing admissions to the pediatric ED due to both RSV and other respiratory virus-related bronchiolitis. Secondary aims included evaluating the impact of nirsevimab on hospitalization rates, describing the clinical course of bronchiolitis and comparing clinical and epidemiological characteristics between immunized and non-immunized patients.

## 2. Materials and Methods

This was a single-center retrospective cohort study conducted through the analysis and comparison of data from all children aged <24 months admitted for bronchiolitis at the tertiary pediatric ED of our Children’s Hospital (accounting for 40,000 admissions/year) during the last two RSV epidemic seasons, between 1 October 2023 and 30 April 2024 and between 1 October 2024 and 30 April 2025, respectively.

The following diagnostic ICD-codes, 10th revision, were considered and retrospectively included: 466.11 (acute bronchiolitis due to RSV) and 466.19 (acute bronchiolitis due to other infectious agents). If the same child was admitted to our ED for bronchiolitis more than once within a 15-day interval, such admissions were considered as part of a single infectious episode, and only data from the more severe episode were analyzed. Otherwise, if two admissions occurred more than 15 days apart, they were considered separate infectious events and were both included in the analysis. Patients older than 24 months, those presenting with upper respiratory tract infections and those discharged with a different diagnosis were excluded. Demographic and clinical data, laboratory results, microbiological and therapeutic data of all patients were extracted from electronic records. Each patient was identified with a progressive numerical code to guarantee anonymity.

For each subject included in the study during both RSV seasons, the following data were collected, when available:

▪ Demographic characteristics: Sex, date of birth and age (in months) at the time of the ED admission.
▪ Clinical features on the first ED evaluation: Vital signs, rhinitis, cough, dyspnea, poor feeding, apnea, and bilateral crackles on lung auscultation; symptoms duration and weight were also recorded.
▪ Personal history: Birth weight, gestational age, perinatal complications, feeding method, vaccination status, comorbidities or complex chronic conditions.
▪ Palivizumab (only for patients from the 2023–2024 RSV season) and nirsevimab immunization status (only for patients from the 2024–2025 RSV season), which was reported on birth medical records provided by caregivers during the visit to the ED.
▪ Nasopharyngeal RSV testing: It was verified whether the test had been performed, and, if so, the result and the date of sample collection were recorded. Results from both antigenic and molecular swabs were considered.
▪ Detection of other respiratory pathogens, including Influenza A, Influenza B, SARS-CoV-2, or other positive viral swabs.
▪ Hospitalization data: Total length of hospital stay, discharge mode, admission to Pediatric Intensive Care Unit (PICU) and total PICU length of stay.
▪ Therapy during the stay in the ED or throughout hospitalization.
▪ Oxygen support requirements, if needed: Nasal cannula, high-flow nasal cannula (HFNC), nasal continuous positive airway pressure (cPAP) or invasive ventilation, along with the total duration of required oxygen therapy.
▪ Nutritional support, if needed: Peripherical venous catheter and/or nasogastric tube.

In both seasons, the identification of the causal viral agent for bronchiolitis was carried out using nasopharyngeal aspirate with an antigen test and/or with multiplex polymerase chain reaction (PCR) assay (FilmArray^®^). The diagnosis of RSV infection was based on a positive RSV nasopharyngeal swab.

Bedside antigenic tests were performed directly in the ED: the STANDARD F2400 analyzer (SD BIOSENSOR, Suwon, Republic of Korea), based on fluorescence-based lateral flow immunoassay technology, was used paired with the RSV Ag FIA test produced by the same company, for the detection of RSV antigens in nasopharyngeal swabs within 15 min. Based on the manufacturer’s clinical evaluation, the sensitivity of the test is 98.11%, and the specificity is 100%.

*Influenza A/B* and *SARS-CoV-2* were detected through bedside antigenic tests performed in the ED on the same analyzer paired with SD BIOSENSOR Standard Q Influenza A&B test and Standard Q COVID/Flu Ag Combo test (SD BIOSENSOR, Suwon, Republic of Korea). The other viruses were detected using a multiplex polymerase chain reaction (PCR) assay (FilmArray^®^ ).

All information was collected in full respect of the children’s and their families’ privacy. In accordance with European regulations, Italian observational studies from data obtained without any additional therapy or monitoring procedures do not need the approval of an Independent Ethical Committee.

Descriptive analysis was performed. Categorical variables were reported as frequencies and percentages, while continuous variables were reported as median and interquartile range (IQR). Differences between groups were assessed using the Chi-Square test for categorical variables, with Fisher’s exact test applied when expected cell frequencies were below five. Continuous variables were analyzed using the Wilcoxon rank-sum non-parametric tests to assess differences in distributions, which, under similar distributional shapes, can be interpreted as differences in medians.

Moreover, a univariable logistic model was designed to evaluate the impact of immunization against bronchiolitis on both RSV at hospitalization and access to PICU. Then, some possible confounders of the association were also included in the multivariable logistic regression models. To compare the effect across seasons, separate logistic regression models were fitted within each seasonal stratum. Potential confounders (age, sex, breastfeeding and weight at birth) were included in the models. The results were reported using Odds Ratio (OR) and 95% confidence intervals (CIs). Significance was set at *p* < 0.05. Statistical analysis was performed using the software R 4.4.2.

## 3. Results

Overall, 484 patients were included: 336 were admitted during the first epidemic season and 148 during the second epidemic season (−56%). In both seasons, the mean age at presentation was similar (3.01 ± 2.12 months vs. 2.91 ± 2.06 months, respectively; *p* = 0.648), males prevailed (51.5% and 53.4%, respectively), and the proportion of infants born at term was comparable. In the first season, associated comorbidities were less frequent than in the second one (6.8% vs. 17.6%; *p* < 0.001), while breastfeeding was more frequently reported (69.3% vs. 53.4%; *p* < 0.001).

Regarding clinical signs and symptoms at presentation, only cough, rhinitis and bilateral crackles on lung auscultation were significantly more frequent during the second epidemic season compared to the first (*p* = 0.028, *p* < 0.001 and *p* = 0.028, respectively). The hospitalization rate was stable.

**Figure 1:**
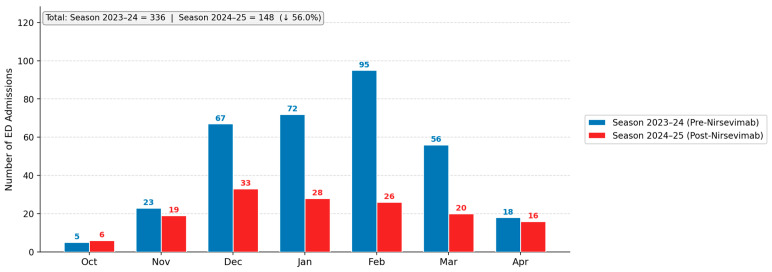
Monthly admissions due to bronchiolitis at the Emergency Department between October 2023 and April 2024 (blue bars) and between October 2024 and April 2025 (red bars).

All the information regarding demographic characteristics, past medical history, vaccinations and nirsevimab status, clinical and care-related characteristics of our population are reported in Table 1. The seasonal bronchiolitis trend by month of admission to the ED is shown in Figure 1.

**Figure 2:**
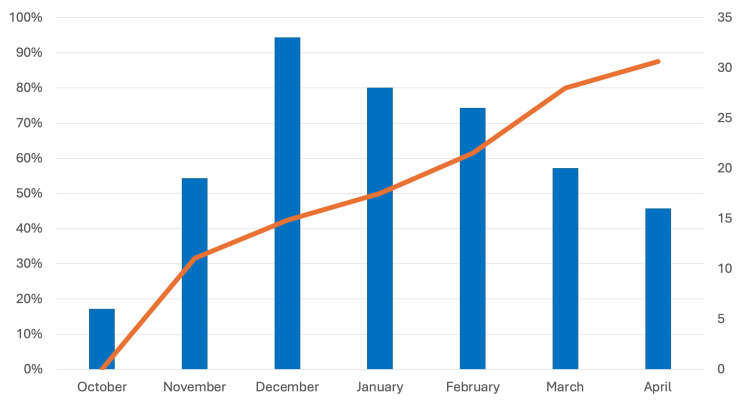
Progressive reduction in monthly admissions due to bronchiolitis at the Emergency Department between October 2024 and April 2025 (blue bars) and concomitant rise in the rate of patients who had received nirsevimab on the total number of patients with bronchiolitis (orange line).

The rate of patients who had received nirsevimab per month ranged from 31.6% in November 2024 to 87.5% in April 2025 (Figure 2).

### 3.1. Nasopharyngeal Swabs Findings

The rate of RSV-positive swabs was 47.9% in the first season and decreased to 32.4% in the second season (*p* = 0.005). Among RSV-positive patients, the rate of co-infections increased significantly from 4.3% in the first season to 14.6% in the second one (*p* = 0.021).

The rates of *Influenza A*, *Influenza B*, and *SARS-CoV-2* did not show significant differences between the two epidemic seasons. Conversely, positive swabs for other viral etiological agents as a whole (*Rhino-Enterovirus*, *Metapneumovirus*, *Adenovirus*, *Coronavirus*) showed a significant rise in the second season, from 3.9% to 20.3% (*p* < 0.001) (Table 2).

### 3.2. Comparison Between Hospitalized Patients in the Two Epidemic Seasons

Out of the total 484 patients, 328 were admitted to general pediatric wards (226 in the first season and 102 in the second one). The rate of RSV-positive hospitalized patients was significantly higher in the first season (64.2% vs. 45.1%; *p* = 0.001), while the proportion of co-infections in RSV-positive patients increased significantly from 3.4% to 15.2% in the second season (*p* = 0.009). Furthermore, positive swabs for other viral agents in RSV-negative patients increased markedly from 4.9% to 28.4% (*p* < 0.001).

Oxygen support by any means was delivered more frequently in the first season (81.9% vs. 72.5%; *p* = 0.056). cPAP support was needed for a shorter time in the second season, decreasing from 2.00 days (IQR 2.00–3.75) to 1.00 day (IQR 1.00–1.00) (*p* = 0.003). Intubation rate did not differ between the two cohorts.

Regarding nutritional support, parenteral hydration was required in approximately half of the patients in both seasons. However, the total duration of hydration was shorter in the first season: 4 days (IQR 3–5) vs. 5 days (IQR 3–6) (*p* = 0.033). Complete details about hospitalized patients are reported in Table 3.

A multivariable logistic regression model was performed to investigate whether the immunization reduced the RSV positivity in the second season. To compare the effect across seasons, separate logistic regression models were fitted within each seasonal stratum. ORs with corresponding 95% CIs are reported in Table 4. Immunization was not associated with RSV positivity in the first epidemic season (OR = 0.842, 95% CI: 0.156–5.059), whereas it was associated with a reduced risk of an RSV-positive swab in the second season (OR = 0.159, 95% CI: 0.059–0.397). These findings are consistent with the lower RSV positivity rates reported in Table 3.

### 3.3. Characteristics of Patients by Nirsevimab Status

Clinical, demographic and care-related characteristics of the 148 pediatric patients admitted to the ED for bronchiolitis during the 2024–2025 epidemic season were analyzed according to their nirsevimab status (Table 5).

Overall, 80 patients (54.1%) had received nirsevimab. Among them, the proportion of RSV-positive patients was significantly lower than in non-immunized patients (16.2% vs. 51.5%, *p* < 0.001).

A higher proportion of males had received nirsevimab compared to females (61.3% vs. 38.8%, respectively; *p* = 0.037). Comparing those who had received nirsevimab to those who had not, the rate of preterm infants was higher (22.8% vs. 10.3%, *p* = 0.044), mean body weight at presentation and mean birth weight were lower (*p* = 0.024 and *p* = 0.012, respectively), and mean peripheral oxygen saturation was higher (*p* = 0.005). There were no differences between the two groups in terms of comorbidities or breastfeeding rates.

No differences were also observed regarding the hospitalization rate, the total length of stay in hospital and clinical complications. Parenteral nutritional support was significantly less frequently required in patients who had received nirsevimab (25% vs. 48.5%, *p* = 0.003).

### 3.4. Characteristics of Hospitalized Patients Among Those Who Had Received Nirsevimab

Among the 80 patients who had received immunization with nirsevimab, 52 (65%) required hospital admission.

In non-hospitalized patients, all tests performed were antigen-based, whereas among hospitalized patients, 40.4% received a molecular swab (*p* < 0.001), to allow for a more comprehensive virologic diagnostic assessment (Table 6).

Among the 52 hospitalized patients, only 13 (25%) tested RSV-positive. In contrast, among the 28 immunized patients with bronchiolitis who were not hospitalized, 15 (53.6%) were RSV-negative; in the remaining 13 patients (46.4%), no nasopharyngeal swab of any type was performed.

Therefore, among children who had received nirsevimab, RSV negativity was observed in 63.5% of hospitalized patients vs. 53.6% of non-hospitalized ones (*p* < 0.001). Positive swabs for other respiratory viruses were reported in 19 hospitalized patients (38.5%) compared to 1 non-hospitalized patient (3.6%) (*p* < 0.001).

### 3.5. Characteristics of Patients Admitted to the Pediatric Intensive Care Unit

A total of 29 children were admitted to PICU over the last two epidemic seasons: 18 in 2023–2024 and 11 in 2024–2025 (Table 7). Distribution by sex and age was similar between the two groups, and the RSV-positivity rate decreased from 88.9% to 63.6%, but this difference did not reach statistical significance (*p* = 0.087).

Comorbidities were infrequent in both groups (5.6% vs. 18.2%), with no significant difference. However, breastfeeding was significantly more frequent in the first season (66.7% vs. 27.3%, *p* = 0.039).

Regarding respiratory support during PICU admission, the median duration of cPAP support was significantly shorter in the second season, decreasing from 2 days to 1 day (*p* = 0.001).

In the second season, five patients (45.5%) had already received nirsevimab, while six (54.5%) had not. Among the five immunized patients, only two tested positive for RSV: one had a co-infection with *Rhino-Enterovirus*, and the other was an extremely preterm infant (gestational age: 26 + 6 weeks).

Regarding the serologic status of all the infants admitted to PICU in the second season, 7/11 (63.6%) tested positive for RSV. Out of them, one showed comorbidity (extreme prematurity), and two were breastfed. Notably, 3/7 RSV-positive infants had a co-infection with both RSV and *Rhino-Enterovirus*. Among the four RSV-negative patients, three tested positive for *Rhino-Enterovirus*, and one was positive for both *Rhino-Enterovirus* and *Metapneumovirus*.

Last, we compared the 29 patients admitted to the PICU in both seasons to the other 455 patients included in this study. The median age of infants admitted to PICU was significantly lower (1 month vs. 3 months, *p* < 0.001), as well as body weight at ED presentation (4608.4 ± 1198.2 g vs. 5713.3 ± 1521.9 g, *p* < 0.001).

The RSV-positive rate was significantly higher in patients admitted to the PICU (79.3% vs. 40.9%; *p* < 0.001). Moreover, RSV-negative patients admitted to the PICU showed a significantly higher prevalence of testing positive for other respiratory viruses (31% vs. 9%, *p* < 0.001).

Results from a multivariable logistic regression model showed that immunization with nirsevimab was associated with a reduced risk of admission to PICU, but such a reduction was not significant (Table 8). The risk of admission to PICU decreased by 35.8% with every additional month of age (OR = 0.642, 95% CI: (0.479–0.827)). Breastfeeding was associated with 55.1% lower risk of admission to PICU, though significance was not reached (OR = 0.449, 95% CI: (0.199–1.089)). Given the small number of PICU admissions, the multivariable model may be affected by overfitting, as the limited number of events relative to the included covariates can lead to unstable estimates and reduced reliability of the associations. In addition, the model showed limited explanatory ability (McFadden pseudo − R^2^ = 0.08). Therefore, these findings should be interpreted with caution and confirmed in larger cohorts.

## 4. Discussion

Our study provides a real-life overview of the impact of nirsevimab prophylaxis within the setting of a large tertiary-level Italian pediatric ED.

Several interesting findings clearly emerge. First, data from our center for the 2024–2025 season show that nirsevimab prophylaxis was associated with a marked reduction in admissions to our ED for both all-cause bronchiolitis and RSV-related bronchiolitis. This is consistent with the results provided by the MELODY and HARMONIE trials [18,19] and by the initial experiences after large-scale implementation [20,22,23,24,25].

A large retrospective cohort study from Catalonia, involving 15,341 infants, reported adjusted hazard ratios of 0.26 for hospital admission (74% reduction), 0.15 for PICU admission (85% reduction), and 0.46 for ED visits (54% reduction) among infants who received nirsevimab compared to unimmunized historical controls [24]. Similarly, a case–control study from France demonstrated significant reductions in ED attendances and subsequent hospitalizations for bronchiolitis following national nirsevimab rollout [20]. A systematic review synthesizing early real-world evidence from the United States, Luxembourg, and Spain reported effectiveness against RSV hospitalization ranging from 82% to 91% in individual studies, with population-level reductions exceeding 60% in regions with high uptake [23]. The consistency of our observed reductions with such cohort studies, despite differences in healthcare settings and study designs, supports the robustness of the protective effect of nirsevimab against severe RSV disease requiring emergency care and hospitalization. The somewhat lower reduction in total bronchiolitis admissions in our study (56%) compared to RSV-specific reductions (70.6%) likely reflects the contribution of non-RSV viral etiologies to bronchiolitis burden, as observed also in other real-world evaluations [23,24].

In the 2024–2025 epidemic season, after the introduction of nirsevimab, the number of ED visits for bronchiolitis peaked in December 2024 and January 2025, as well as the number of RSV-positive patients. This seasonal peak was expected and was consistent with the literature [7], suggesting a return to the pre-SARS-CoV-2 pandemic epidemiological pattern [8,9,10,11]. The COVID-19 pandemic and associated nonpharmaceutical interventions profoundly disrupted RSV circulation, leading to altered seasonality and potential changes in disease severity that have implications for immunoprophylaxis timing and patient selection. Following relaxation of pandemic restrictions, RSV epidemics resurged with atypical timing, including earlier season starts, off-season peaks, and variable epidemic duration [25,26,27]. These shifts in seasonality necessitate flexible prophylaxis schedules informed by real-time surveillance rather than fixed calendar-month administration to maximize protection during emergent peaks [25,26].

Evidence regarding post-pandemic disease severity is mixed but suggests potential increases in certain cohorts. The prospective PAPI study, comparing hospitalized RSV patients under 2 years of age before and after the pandemic, found significantly higher rates of oxygen supplementation and non-invasive ventilation in the post-pandemic period, suggesting increased severity among hospitalized cases [26]. A two-season comparative study similarly documented shifts in clinical presentation and severity markers during post-pandemic RSV seasons [28]. These findings may reflect reduced prior exposure in infant cohorts born during pandemic restrictions, leading to greater susceptibility and potentially more severe disease upon first RSV encounter.

It is noteworthy that in our population, the absolute number of hospital admissions for bronchiolitis in the 2024–2025 season was almost halved compared to the previous season. Nevertheless, nirsevimab was not associated with any modification of the hospitalization rate for all-cause bronchiolitis or RSV-associated bronchiolitis. This may be attributable to two main factors: on one hand, the limited sample size of our single-center study; on the other hand, this was only the first year of the prevention campaign, and a consistently high immunization rate has been reached only in the last months of the RSV epidemic season. We can argue that an earlier start of the campaign in future seasons, or even continuous prevention with nirsevimab, could positively affect the hospitalization rate as well. Moreover, with a larger sample size and a consistently high immunization rate, a clear decrease in hospitalization rates could be observed, as reported in Valle d’Aosta Italian region during the 2023–2024 epidemic season and in other European countries, such as Spain, France, Germany and the United Kingdom [19,29,30,31].

Regardless, despite the results regarding hospitalization rates, our data already show a robust association between nirsevimab prophylaxis, lighter clinical presentation and disease course, and better prognosis for infants with bronchiolitis. Indeed, immunized RSV-positive patients showed milder disease compared to non-immunized patients, and the severity of bronchiolitis was often attributable to co-infections. We observed a reduction in the duration of cPAP support among hospitalized patients, suggesting less severe respiratory impairment. Furthermore, parenteral hydration was markedly reduced in immunized patients compared to non-immunized ones, as well as the incidence of complications. Such findings suggest that, on one hand, nirsevimab has reduced the disease incidence and, on the other hand, it could have contributed to attenuating its severity, improving both the clinical course and prognosis of those cases that still required hospitalization, despite not yet modifying the crude hospitalization rate. This is consistent with the reduction in the severe disease rate documented both in clinical trials [19] and in major real-life studies [29].

The overall immunization rate observed in our study was 54.1%. At first glance, this observation differs partially from the literature. Higher immunization rates to date have been reported in Spain [31], achieving 90.1% during the 2023–2024 season, followed by France [32] and Luxembourg [33], both reporting rates close to 85%. Alternatively, during the 2023–2024 season in the United States, the immunization rate was only 51.2% [34]. The United Kingdom implemented universal maternal vaccination as a national program for 2024–2025, despite nirsevimab currently being recommended only for high-risk children; consequently, there is no universally comparable national infant coverage rate. As for Italy, reliable immunization rate data are available for the Valle d’Aosta region, where coverage was 86%, as well as the Piedmont (71.5%) and Sicily (below 70%) regions [22,29,35].

The apparently low immunization rate observed in our study warrants several considerations. First, this study examined only the first RSV epidemic season in which the immunization campaign was launched, a period characterized by initial logistical challenges and, at times, shortages of the medication. Second, the reported immunization rate of 54.1% represents the mean across all seven months analyzed in the study. When examining the monthly rates, it is striking to note the progression from 31.6% in November 2024—when nirsevimab was introduced—to 87.5% in April 2025, as the immunization campaign became increasingly systematic and widely implemented. This suggests that if the immunization program remains equally well-structured in the upcoming RSV seasons, coverage rates in our region could be consistently similar to those observed in Spain. However, implementation heterogeneity can significantly limit program effectiveness and exacerbate health inequities. Achieving the large reductions in ED presentations and hospitalizations observed in high-performing cohorts requires uniform, timely distribution and high coverage.

Nirsevimab represents a significant advance over palivizumab for RSV immunoprophylaxis, with important implications for program design, population impact, and cost-effectiveness. Unlike palivizumab, which requires monthly intramuscular injections throughout the RSV season and has historically been restricted to high-risk infants, the extended half-life of nirsevimab enables single-dose protection for an entire RSV season, facilitating universal immunoprophylaxis strategies [23,36,37].

Cost-effectiveness modeling from multiple countries supports universal nirsevimab strategies under plausible acquisition prices. A Netherlands analysis estimated that universal nirsevimab could prevent thousands of RSV hospitalizations annually [36]. Japanese modeling projected that universal nirsevimab could reduce approximately 50% of RSV-associated health events and be cost-effective at modeled prices [38]. An Italian cost-effectiveness analysis similarly supported all-infant nirsevimab strategies under certain price thresholds [39]. In contrast, palivizumab was not found to be cost-effective for certain preterm groups in US modeling when compared to no prophylaxis [40]. However, realized cost-effectiveness depends critically on actual acquisition costs, program coverage, and timely, uniform implementation, as demonstrated by the heterogeneous outcomes of early rollout experiences [6,36,38].

Overall, immunization was associated with a marked reduction in the proportion of RSV-positive patients in our ED. Consequently, the proportion of RSV-positive swabs declined substantially among patients admitted both to general wards and PICU.

Among immunized patients, 60% tested RSV-negative. The clearest signal of nirsevimab’s beneficial impact on RSV infection was the markedly lower RSV positivity rate among immunized patients compared to non-immunized ones. Furthermore, immunized infants presented higher mean oxygen saturation on admission, less frequent parenteral hydration and fewer complications. All these findings suggest a milder disease profile among immunized individuals.

In the 2024–2025 epidemic season, alongside the overall reduction in RSV-positive rate, there was a significant increase in positive swabs for other respiratory viruses, such as human *Metapneumovirus*, *Rhinovirus/Enterovirus* and *Adenovirus*. This observation requires careful interpretation, as multiple alternative explanations must be considered before attributing this shift to any single factor.

First, the post-COVID-19 pandemic period has been characterized by substantial alterations in respiratory virus epidemiology across multiple settings. Non-pharmaceutical interventions adopted during 2020–2021 reduced childhood exposures to endemic respiratory viruses, creating larger cohorts of susceptible children—a phenomenon termed “immunity debt” [41,42]. As restrictions were relaxed, numerous studies documented off-season and intensified waves of *Rhinovirus*, *Metapneumovirus*, *Adenovirus*, and other non-RSV pathogens [41,43,44]. These post-pandemic rebound dynamics may continue to influence viral circulation patterns during our study period (2023–2025), independent of nirsevimab introduction.

Second, changes in testing practices and surveillance intensity may substantially affect observed case counts. The pandemic era brought widespread adoption of multiplex PCR panels and increased testing volume in pediatric settings. A multicenter analysis demonstrated that over two-thirds of the apparent rise in RSV case counts and age distribution shifts were attributable to increased testing rather than increased circulation alone [45]. The enhanced detection of non-RSV viruses through more sensitive diagnostic platforms and broader testing indications may contribute to the higher detection rates observed in our second season, particularly for milder cases that might have previously gone undiagnosed.

Third, respiratory viruses exhibit substantial natural interannual variability in timing, intensity, and dominant circulating strains [46,47,48]. Time-series analyses demonstrate pathogen-specific seasonal fluctuations that existed pre-pandemic and were further perturbed by NPIs. Some of the observed changes in our study may reflect natural seasonal variation rather than a sustained epidemiological shift.

Fourth, viral interference and competitive dynamics among co-circulating respiratory viruses can alter epidemic patterns through immunological and ecological mechanisms [49,50,51]. The differential impact of NPIs on various respiratory pathogens and their subsequent rebound may have altered competitive relationships at the population level. However, these dynamics are complex and influenced by multiple factors beyond any single intervention.

Finally, while nirsevimab was associated with reduced RSV circulation in our cohort (RSV positivity decreased from 47.9% to 32.4%, *p* = 0.003), its potential role in the rise in non-RSV viruses remains speculative. Ecological theory suggests that reducing one dominant pathogen could create opportunities for others through reduced viral interference or altered host susceptibility patterns [49,50]. However, establishing such a causal relationship would require controlled studies, community-level surveillance, and mechanistic investigation. Any potential contribution of nirsevimab to non-RSV virus increases would likely be modest and operate in conjunction with the multiple factors outlined above.

Our logistic regression models showed a protective trend of nirsevimab against severe outcomes and the risk of PICU admission; however, the lack of statistical significance prevents extending our findings to the general population. This may reflect the small sample size from a single season and the limited number of events, rather than a lack of efficacy. We can suppose that, with a larger sample size, this finding might reach significance, in line with the literature [19,20,30,32]. Conversely, age had a clear protective effect against PICU admission, with older children showing a progressively lower risk.

Our study provides an overall evaluation of the impact of nirsevimab on children with bronchiolitis admitted to the pediatric ED. In the 2024–2025 cohort, they showed a higher prevalence of comorbidities compared to the previous season. Moreover, immunized infants were more frequently preterm and had lower birth weight, suggesting that the prevention campaign largely succeeded in reaching at-risk and vulnerable infants due to prematurity.

As previously noted, the infants of the second epidemic season were less frequently RSV-positive and showed more favorable clinical predictors. Among immunized children, RSV positivity dropped markedly, leaving non-immunized children comparatively more vulnerable; indeed, they were more frequently RSV-positive and showed a higher rate of bronchiolitis-associated complications.

The interpretation of the observed epidemiological changes following the introduction of nirsevimab should be approached with caution due to potential temporal and ecological bias. Temporal trends unrelated to the intervention, including pre-existing fluctuations in RSV circulation and the disruption and subsequent re-establishment of RSV transmission patterns, caused by the COVID-19 pandemic, may have altered both the age distribution and susceptibility of pediatric populations. Additionally, changes over time in testing practices, healthcare-seeking behavior, and hospital admission criteria could have contributed to variations in reported incidence and severity.

From an ecological perspective, analyses based on aggregated data are inherently limited by the risk of ecological fallacy, as population-level associations do not necessarily reflect individual-level effects. Heterogeneity in nirsevimab uptake across regions and risk groups, along with unmeasured confounders such as underlying comorbidities and socio-demographic factors, may further bias the findings. Furthermore, potential indirect effects on viral transmission dynamics could contribute to changes in disease burden independently of direct protection among treated individuals.

This study has some strengths. First, the completeness and accuracy of clinical and management data collection. Clinical presentation indicators, microbiology results, and detailed in-hospital care pathways were analyzed and integrated, specifying all types and intensities of respiratory and nutritional support, therapeutic approaches, and a thorough analysis of complications and overall hospital admissions, both to general pediatric wards and to PICU. This allowed for a precise and reliable multidimensional assessment of the impact of immunization on admissions for bronchiolitis to our pediatric ED.

Second, by encompassing the last two RSV epidemic seasons, the study design enabled a direct and exhaustive comparison between the pre- and post-nirsevimab eras, facilitating the evaluation of the impact of immunization, not only on admissions and hospitalizations, but also on disease severity and in-hospital management.

However, our study also has some limitations. First, this is a retrospective study with a limited sample size, which results in insufficient statistical power for some endpoints; also, the lack of adjustment for confounders represents a limitation. Second, the analysis of only the first year after the introduction of nirsevimab does not allow for assessment of long-term effects or potential seasonal variations in subsequent years. Furthermore, the single-center design limits the ability to capture possible differences in admission and hospitalization criteria across other settings, thereby restricting the generalizability of our results to a broader population, along with the potential variability of RSV epidemiology across different seasons and regions. Last, by focusing solely on a tertiary pediatric ED, there is an inherent selection bias: immunized children who become ill and require hospital evaluation do not represent a random sample of the entire immunized pediatric population. Moreover, we must consider that our ED allows both direct admissions and referrals of the most severe cases from community hospitals throughout our region. This could reflect a higher representation of fragile infants in our population compared to those accessing community hospitals.

From this analysis, many future directions, practical implications and recommendations emerge. First and foremost, it is essential to conduct multicenter and multi-seasonal studies to provide more robust evidence on the overall efficacy of nirsevimab.

It is equally crucial to invest as many resources as possible in consolidating and expanding the prevention campaign. Indeed, the case for universal RSV prophylaxis is further reinforced by several European experiences, which show that coverage rates above 80–90% are associated with a marked reduction in admissions to both the general ward and the PICU [29,32,33,34]. Furthermore, it is necessary to integrate broad-spectrum virologic surveillance, given the increase in other respiratory viruses and the viral shifts recorded in the last epidemic season.

## 5. Conclusions

Our study showed that RSV prevention through nirsevimab prophylaxis was associated with lower RSV-positivity rates and a trend toward reduced clinical severity.

The high incidence of viral co-infections in the second season, particularly with Rhino-Enterovirus, highlights the role of other respiratory pathogens in determining symptoms and clinical outcomes.

The observed rise in non-RSV viral detections in our hospital-based cohort is likely multifactorial, reflecting a complex interplay of post-pandemic immunity debt, altered testing practices, natural seasonal variability, viral competitive dynamics, and selection bias inherent to hospital-based surveillance. While the impact of nirsevimab on RSV circulation is clear, attributing changes in non-RSV viral epidemiology to this intervention alone would be premature. Future research should incorporate community-based surveillance, standardized testing protocols across seasons, and serological studies to disentangle these competing explanations and better understand the broader ecological consequences of RSV immunoprophylaxis programs.

Overall, our results support the role of nirsevimab in universal prevention programs and underscore the need for further prospective multicenter studies to confirm the long-term impact of this preventive strategy on morbidity and healthcare resource utilization.

## Figures and Tables

**Table 1:** Demographic characteristics, past medical history, vaccinations, nirsevimab status, and clinical and care-related characteristics of the study population.

		2023–2024 RSV Epidemic Season 1 October 2023–30 April 2024	2024–2025 RSV Epidemic Season 1 October 2024–30 April 2025	*p*-Value
Overall (*n* = 484)		336	148	
Age, months	Median (IQR)	3 (1–4)	3 (1–4)	0.833
Sex	FemaleMale	163 (48.5) 173 (51.5)	69 (46.6) 79 (53.4)	0.701
Weight at birth, grams	Mean (SD)	3087.84 (573.72)	2976.48 (654.34)	0.075
Born at term (≥37 weeks)	Yes No	289 (86.0) 47 (14.0)	123 (83.0) 25 (17.0)	0.391
Comorbidities	Yes No	23 (6.8) 313 (93.2)	26 (17.6) 122 (82.4)	**<0.001**
Breastfeeding	Yes No	233 (69.3) 103 (30.7)	79 (53.4) 69 (46.6)	**0.001**
Vaccinations	Yes No	171 (50.9) 165 (49.1)	91 (61.5) 57 (38.5)	**0.031**
Palivizumab	Yes No	8 (2.4) 68 (97.6)	NA NA	NA NA
Nirsevimab	Yes No	NA NA	80 (54.1) 68 (45.9)	NA NA
Weight at ED presentation, grams	Median (IQR)	5560.0 (4500.0–6612.5)	5450.0 (4650.0–6725.0)	0.734
Peripheral oxygen saturation	Mean (SD)	95.76 (3.77)	95.53 (3.59)	0.534
Respiratory rate, breaths per minute	Median (IQR)	54.0 (46.00–60.00)	52.00 (45.75–60.00)	0.958
Temperature ≥ 38 °C	Yes No	82 (24.4) 254 (75.6)	25 (16.9) 123 (86.1)	0.066
Cough	Yes No	296 (88.1) 40 (11.9)	140 (94.6) 8 (5.4)	**0.028**
Rhinitis	Yes No	201 (59.8) 135 (40.2)	136 (91.9) 12 (8.1)	**<0.001**
Dyspnea	Yes No	245 (72.9) 91 (27.1)	118 (79.7) 30 (20.3)	0.111
Bilateral crackles	Yes No	194 (57.6) 142 (42.4)	101 (68.2) 47 (31.8)	**0.027**
Hospitalization	Yes No	226 (67.3) 110 (32.7)	102 (68.9) 46 (31.1)	0.719
Oxygen Support, total days	Median (IQR)	5 (3–7)	5 (3–7)	0.777
Occurrence of complications	Yes No	52 (15.5) 284 (84.5)	31(20.9) 117 (79.1)	0.141
Admission to PICU	Yes No	18 (5.4) 318 (94.6)	11 (7.4) 137 (92.6)	0.380

Abbreviations: IQR = interquartile range, SD = standard deviation, ED = Emergency Department, PICU = Pediatric Intensive Care Unit.

**Table 2:** Results of nasopharyngeal swabs in both epidemiological seasons.

		2023–2024 RSV Epidemic Season 1 October 2023–30 April 2024	2024–2025 RSV Epidemic Season 1 October 2024–30 April 2025	*p*-Value
Overall (*n* = 484)		336	148	
RSV	Positive Negative Not Performed	161 (47.9) 105 (31.2) 70 (20.8)	48 (32.4) 65 (43.9) 35 (23.6)	**0.005**
Co-Infections with RSV *	Yes No	7 (4.3) 154 (95.7)	7 (14.6) 41 (85.4)	**0.021**
*Influenza A*	Positive Negative	3 (0.9) 333 (99.1)	2 (1.4) 146 (98.6)	0.644
*Influenza B*	Positive Negative	0 (0.0) 336 (100.0)	2 (1.4) 146 (98.6)	0.093
*SARS-CoV-2*	Positive Negative	4 (1.2) 332 (98.8)	0 (0.0) 148 (100.0)	0.318
Other positivity detected	Yes No Not detected	13 (3.9) 4 (1.2) 319 (94.9)	29 (20.3) 10 (6.8) 109 (73.0)	**<0.001**
Type of positivity	*Metapneumovirus* *Rhino/Enterovirus* *Rhino/Enterovirus + Metapneumovirus* *Rhino/Enterovirus + Adenovirus* *Coronavirus*	5 (38.5) 6 (46.2) 2 (15.4) 0 (0.0) 0 (0.0)	5 (17.2) 17 (58.6) 2 (6.9) 3 (10.3) 2 (6.9)	0.363

* The sample size refers only to RSV-positive patients.

**Table 3:** Characteristics of hospitalized patients in both epidemiological seasons.

		2023–2024 RSV Epidemic Season 1 October 2023–30 April 2024	2024–2025 RSV Epidemic Season 1 October 2024–30 April 2025	*p*-Value
Overall (*n* = 328)		226	102	
Length of hospitalization	Median (IQR)	7 (5–9)	6 (4–10)	0.873
Nirsevimab	Yes No	NA NA	50 (49.0) 52 (51.0)	NA NA
RSV	Positive Negative Not Performed	145 (64.2) 72 (31.9) 9 (4.0)	46 (45.1) 44 (43.1) 12 (11.8)	**0.001**
Co-Infections with RSV *	Yes No	5 (3.4) 140 (96.6)	7 (15.2) 39 (84.8)	**0.009**
Other positivity detected	Yes No	11 (4.9) 215 (95.1)	29 (28.4) 73 (71.6)	**<0.001**
Oxygen support	Yes No	185 (81.9) 41 (18.1)	74 (72.5) 28 (27.5)	0.056
Oxygen support, total days	Median (IQR)	5 (3–7)	5 (3–7)	0.777
Nasal cannula	Yes No	114 (50.4) 112 (49.6)	59 (57.8) 43 (42.2)	0.164
Total duration of nasal cannula, in days	Mean (SD)	2.54 (2.05)	2.74 (2.10)	0.607
HFNC	Yes No	114 (50.4) 112 (49.6)	46 (45.1) 56 (54.9)	0.370
Total duration of HFNC, in days	Median (IQR)	6.00 (4.00–7.00)	5.00 (5.00–7.00)	0.822
cPAP	Yes No	15 (6.6) 211 (93.4)	12 (11.8) 90 (88.2)	0.118
Total duration of cPAP, in days	Mean (SD) Median (IQR)	2.86 (1.92) 2.00 (2.00–3.75)	1.33 (0.89) 1.00 (1.00–1.00)	**0.015** **0.003**
Invasive ventilation	Yes No	3 (1.3) 223 (98.7)	2 (2.0) 100 (98.0)	0.648
Total duration of invasive ventilation, in days	Median (IQR)	3.00 (2.50–6.50)	7.50 (7.25–7.75)	0.564
PVC	Yes No	126 (56.0) 100 (44.0)	51 (50.0) 51 (50.0)	0.313
Total duration of PVC, in days	Median (IQR)	4 (3–5)	5 (3–6)	**0.033**
Nasogastric tube	Yes No	22 (9.7) 204 (90.3)	15 (14.7) 87 (85.3)	0.188
Occurrence of complications	Yes No	50 (22.1) 176 (77.9)	31 (30.4) 71 (69.6)	0.108
Admission to PICU	Yes No	18 (8.0) 208 (92.0)	11 (10.8) 91 (89.2)	0.412

Abbreviations: IQR = interquartile range, SD = standard deviation, NA = not available, HFNC = high-flow nasal cannula, cPAP = continuous positive airway pressure, PVC = peripheral venous catheter, PICU = Pediatric Intensive Care Unit. * The sample size refers only to RSV-positive patients.

**Table 4:** Risk factors associated with RSV positivity among those hospitalized in both seasons (multivariable logistic regression models results).

	Variable	2023–2024 Season Odds Ratio (95% Confidence Interval)	2024–2025 Season Odds Ratio (95% Confidence Interval)
Immunization (Palivizumab and Nirsevimab)	No Yes	Reference Category 0.842 (0.156–5.059)	Reference Category **0.159 (0.059–0.397)**
Age		0.929 (0.819–1.503)	0.845 (0.663–1.061)
Sex	Male Female	Reference Category 1.100 (0.626–1.933)	Reference Category 1.094 (0.442–2.669)
Breastfeeding	No Yes	Reference Category 1.532 (0.815–2.873)	Reference Category 0.894 (0.351–2.245)
Comorbidities	No Yes	Reference Category 0.851 (0.362–2.042)	Reference Category 0.924 (0.219–3.595)
Weight at birth	<2500 g ≥2500 g	1.989 (0.782–5.639) Reference Category	0.861 (0.198–3.503) Reference Category

**Table 5:** Characteristics of patients compared by nirsevimab status.

		Did Not Receive Nirsevimab	Received Nirsevimab	*p*-Value
Overall (*n* = 148)		68	80	
Age, months	Median (IQR)	3 (1–4)	3 (1–4)	0.679
Sex	Female Male	38 (55.9) 30 (44.1)	31 (38.8) 49 (61.3)	**0.037**
Weight at birth, grams	Mean (SD) Median (IQR)	3121.16 (593.34) 3125.0 (2970.0–3412.5)	2853.50 (681.76) 3005.0 (2712.5–3225.0)	**0.012** **0.025**
Born at term (≥37 weeks)	Yes No	61 (87.7) 7 (10.3)	62 (77.2) 18 (22.8)	**0.044**
Comorbidities	Yes No	12 (17.6) 56 (82.4)	14(17.5) 66 (82.5)	0.981
Breastfeeding	Yes No	38 (55.9) 30 (44.1)	41 (51.2) 39 (48.8)	0.573
RSV	Positive Negative Not Performed	35 (51.5) 17 (25.0) 16 (23.5)	13 (16.2) 48 (60.0) 19 (23.8)	**<0.001**
Co-Infections with RSV *	Yes No	5 (14.3) 30 (85.7)	2 (15.4) 11 (84.6)	1.000
Weight at ED presentation, grams	Mean (SD) Median (IQR)	6008.31 (1610.86) 5800.00 (5000.00–6943.75)	5432.94 (1431.80) 5187.50 (4537.50–6500.00)	**0.024** **0.040**
Peripheral oxygen saturation	Mean (SD) Median (IQR)	94.62 ± 4.03 96.00 (92.75–97.00)	96.31 ± 2.98 97.00 (95.00–98.25)	**0.005** **0.005**
Hospitalization	Yes No	50 (73.5) 18 (26.5)	52 (65.0) 28 (35.0)	0.264
Length of hospitalization	Median (IQR)	7.00 (4.00–9.75)	6.00 (3.00–10.00)	0.266
Oxygen support	Yes No	39 (57.4) 29 (42.6)	35 (43.8) 45 (56.2)	0.099
Total duration of oxygen support	Median (IQR)	2.0 (0.0–6.0)	0.0 (0.0–5.0)	0.065
Invasive ventilation	Yes No	2 (2.9) 66 (97.1)	0 (0.0) 80 (100.0)	0.209
Peripheral venous catheter	Yes No	33 (48.5) 35 (51.5)	20 (25.0) 60 (75.0)	**0.003**
Nasogastric tube	Yes No	7 (10.3) 61 (89.7)	8 (10.0) 72 (90.0)	0.953
Occurrence of complications	Yes No	19 (27.9) 49 (72.1)	12 (15.0) 68 (85.0)	0.054
Admission to PICU	Yes No	6 (8.8) 62 (91.2)	5 (6.2) 75 (93.8)	0.552
Length of PICU hospitalization	Median (IQR)	4.0 (2.0–6.0)	5.0 (3.0–7.5)	0.447

Abbreviations: IQR = interquartile range, SD = standard deviation, ED = Emergency Department, PICU = Pediatric Intensive Care Unit. * The sample size refers only to RSV-positive patients.

**Table 6:** Characteristics of nirsevimab-immunized patients by need for hospitalization.

		Not Hospitalized	Hospitalized	*p*-Value
Overall (*n* = 80)		28	52	
Length of hospitalization	Median (IQR)	NA	6 (3–10)	
Type of nasopharyngeal test	Antigen-based Molecular (PCR)	28 (100.0) 0 (0.0)	31 (59.6) 21 (40.4)	**<0.001**
RSV	Positive Negative Not Performed	0 (0.0) 15 (53.6) 13 (46.4)	13 (25.0) 33 (63.5) 6 (11.5)	**<0.001**
Co-Infections with RSV *	Yes No	0 0	2 (15.4) 11 (84.6)	NA NA
*Influenza A*	Positive Negative	0 (0.0) 28 (100.0)	2 (3.8) 50 (96.2)	
*Influenza B*	Positive Negative	0 (0.0) 28 (100.0)	1 (1.9) 51 (98.1)	1.000
*SARS-CoV-2*	Positive Negative	0 (0.0) 28 (100.0)	0 (0.0) 52 (100.0)	
Other positivity detected	Yes No Not Detected	1 (3.6) 0 (0.0) 27 (96.4)	19 (38.5) 3 (3.8) 30 (57.7)	**<0.001**
Type of positivity	*Metapneumovirus* *Rhino/Enterovirus* *Rhino/Enterovirus + Metapneumovirus* *Rhino/Enterovirus + Adenovirus* *Coronavirus*	0 (0.0) 1 (100.0) 0 (0.0) 0 (0.0) 0 (0.0)	5 (26.3) 9 (47.4) 2 (10.5) 1 (5.3) 2 (10.5)	
Comorbidities	Yes No	2 (7.1) 26(92.9)	12 (23.1) 40 (76.9)	0.121
Born at term (≥37 weeks)	Yes No	23 (82.1) 5 (17.9)	39 (74.5) 13 (25.5)	0.439
Weight at birth, grams	Median (IQR)	3015.0 (3000.0–3200.0)	2995.0 (2437.5–3252.5)	0.363
History of perinatal complications	Yes No	5 (17.9) 23 (82.1)	11 (21.2) 41 (78.8)	0.725
Admission to PICU	Yes No	0 (0.0) 0 (0.0)	5 (9.6) 47 (90.4)	1.000

Abbreviations: IQR = interquartile range, PCR = protein chain reaction, PICU = Pediatric Intensive Care Unit. * The sample size refers only to RSV-positive patients.

**Table 7:** Characteristics of infants admitted to Pediatric Intensive Care Unit (PICU) in both seasons.

		2023–2024 RSV Epidemic Season 1 October 2023–30 April 2024	2024–2025 RSV Epidemic Season 1 October 2024–30 April 2025	*p*-Value
Overall (*n* = 29)		18	11	
Sex	Female Male	10 (55.6) 8 (44.4)	5 (45.5) 6 (54.5)	0.597
Age, months	Median (IQR)	1.5 (1.0–2.0)	1.0 (1.0–3.5)	0.668
Weight, grams	Median (IQR)	4600.0 (3760.0–5552.5)	4850.0 (3760.0–5100.0)	0.669
Born at term (≥37 weeks)	Yes No	14 (77.8) 4(22.2)	8 (72.7) 3 (27.3)	1.000
Weight at birth, grams	Median (IQR)	3180.0 (3000.0–3585.0)	2980.0 (2695.0–3585.0)	0.5145
Comorbidities	Yes No	1 (5.6) 17 (94.4)	2 (18.2) 9 (81.8)	0.539
Breastfeeding	Yes No	12 (66.7) 6 (33.3)	3 (27.3) 8 (72.7)	**0.039**
Vaccinations	Yes No	2 (11.1) 16 (88.9)	5 (45.5) 6 (54.5)	0.071
Nirsevimab	Yes No	NA NA	5 (45.5) 6 (54.5)	NA NA
RSV	Positive Negative Not Performed	16 (88.9) 1 (5.6) 1 (5.6)	7 (63.6) 4 (36.4) 0 (0.0)	0.087
Length of PICU hospitalization	Median (IQR)	4.0 (2.0–6.0)	5.0 (3.0–7.5)	0.447
Oxygen support	Yes No	17 (94.4) 1 (5.6)	11 (100.0) 0 (0.0)	1.000
Nasal cannula	Yes No	4 (22.2) 14 (77.8)	2 (18.2) 9 (81.8)	1.000
Total duration of nasal cannula, in days	Median (IQR)	1.50 (1.00–2.75)	2.00 (1.50–2.50)	1.000
HFNC	Yes No	18 (100.0) 0 (0.0)	11 (100.0) 0 (0.0)	
Total duration of HFNC, in days	Median (IQR)	5.0 (4.0–7.0)	5.0 (5.0–7.5)	0.3134
cPAP	Yes No	12 (66.7) 6 (33.3)	11 (100.0) 0 (0.0)	**0.058**
Total duration of cPAP, in days	Mean (SD) Median (IQR)	2.55 (1.29) 2.0 (2.0–3.5)	1.36 (0.92) 1.0 (1.0–1.0)	**0.024** **0.001**
Invasive ventilation	Yes No	3 (16.7) 15 (83.3)	2 (18.2) 9 (81.8)	1.000
Total duration of invasive ventilation, in days	Median (IQR)	3.00 (2.50–6.50)	7.50 (7.25–7.75)	0.534
PVC	Yes No	17 (94.4) 1 (5.6)	11 (100.0) 0 (0.0)	1.000
Nasogastric tube	Yes No	12 (66.7) 6 (33.3)	7 (63.6) 4 (36.4)	1.000
Occurrence of complications	Yes No	11 (61.1) 7 (38.9)	7 (63.6) 4 (36.4)	1.000

Abbreviations: IQR = interquartile range, SD = standard deviation, PICU = Pediatric Intensive Care Unit, HFNC = high-flow nasal cannula, cPAP = continuous positive airway pressure, PVC = peripheral venous catheter.

**Table 8:** Risk factors associated with admission to the Pediatric Intensive Care Unit in both seasons (multivariable logistic regression model results).

	Variable	Odds Ratio (95% Confidence Interval)
Immunization (Palivizumab and Nirsevimab)	No Yes	Reference Category 0.703 (0.214–1.916)
Age		**0.642 (0.479–0.827)**
Sex	Male Female	Reference Category 1.086 (0.494–2.398)
Breastfeeding	No Yes	Reference Category 0.449 (0.199–1.019)
Comorbidities	No Yes	Reference Category 0.766 (0.197–2.385)
Born at term (≥37 weeks)	No Yes	Reference Category 0.411 (0.127–1.488)
Weight at birth	<2500 g ≥2500 g	1.060 (0.250–4.047) Reference Category

## Data Availability

The collected data are available from the corresponding author upon reasonable request.

## Abbreviations

The following abbreviations are used in this manuscript:

cPAP	Continuous positive airway pressure
ED	Emergency Department
HFNC	High-flow nasal cannula
IQR	Interquartile range
NC	Nasal cannula
PICU	Pediatric Intensive Care Unit
PVC	Peripheral venous catheter
RSV	*Respiratory syncytial virus*
SD	Standard deviation
